# In Patients with Grade I and II Ankle Sprains, Dynamic Taping Seems to Be Helpful during Certain Tasks, Exercises and Tests in Selected Phases of the Rehabilitation Process: A Preliminary Report

**DOI:** 10.3390/ijerph19095291

**Published:** 2022-04-27

**Authors:** Łukasz Pawik, Malwina Pawik, Emilia Wysoczańska, Aleksandra Schabowska, Piotr Morasiewicz, Felicja Fink-Lwow

**Affiliations:** 1 Department of Physiotherapy in Motor Disorders and Dysfunctions, Faculty of Physiotherapy, Wroclaw University of Health and Sport Sciences, 51-612 Wrocław, Poland; lukasz.pawik@awf.wroc.pl (Ł.P.); emilia.wysoczanska@awf.wroc.pl (E.W.); aleksandra.schabowska@awf.wroc.pl (A.S.); 2 Department of Physiotherapy in Surgical Medicine and Oncology, Faculty of Physiotherapy, Wroclaw University of Health and Sport Sciences, 51-612 Wrocław, Poland; 3 Institute of Medical Sciences, Department of Orthopaedic and Trauma Surgery, University Hospital in Opole, University of Opole, 45-401 Opole, Poland; morasp@poczta.onet.pl; 4 Department of Massage and Physical Therapy, Faculty of Physiotherapy, Wroclaw University of Health and Sport Sciences, 51-612 Wrocław, Poland; felicja.lwow@awf.wroc.pl

**Keywords:** dynamic tape, ankle sprain, kinesiology taping, postural stability, rehabilitation

## Abstract

We aimed to investigate changes in postural stability on a stable surface after the application of dynamic tape for patients with inversion ankle sprains. This study enrolled 30 patients (age 25.5 ± 8.0 years) with grade I and II ankle sprains, which occurred 7–21 days before enrolment. Postural stability (balance, coordination, feedback) was assessed before and after the application of dynamic tape using a stabilographic platform. Three 32-s exercises were performed on the stabilographic platform, one with eyes open, one with eyes closed and one with visual feedback. After the application of dynamic tape, an improvement was observed in terms of the mean radius of sway (4.2 ± 1.3 mm vs. 3.4 ± 0.9 mm; *p* = 0.012) and coordination (48.8 ± 19.2% vs. 59.3 ± 5.8%; *p* = 0.021). Selected balance parameters did not improve significantly in the tests with open and closed eyes. Asymmetric load improved for all tests, but significant differences were only observed with eyes closed (34.9 ± 24.4 vs. 41.7 ± 30.5; *p* < 0.01). We concluded that the use of dynamic tape after an ankle sprain significantly improved balance and coordination on a stable surface. The benefits were shown in terms of a significant improvement in the asymmetric load of the injured limb in comparison to the healthy limb during the test with closed eyes and a considerable improvement in the asymmetric load that was evaluated with visual feedback on a stable surface.

## 1. Introduction

Epidemiological studies have confirmed the systematic increase in the prevalence of inversion ankle sprains, especially among the elderly. Although its prevalence increases with age, this phenomenon is also caused by lifestyle factors, such as sedentary lifestyles, low levels of physical activity and being overweight [1]. These problems are universal and are observed regardless of age or gender. In children who are older than 10 years of age, the frequency of ankle sprains is higher compared to younger children. In turn, between the ages of 15–24 years, this injury is observed more often in men. Women are more often affected after the age of 30 years, which may be due to the improper selection of shoes. It has also been shown that 50% of ankle sprains occur during sports activities [2]. In the US, between 2002 and 2006, the annual reports from emergency departments recorded that 2/1000 patients had ankle injuries [2]. These facts show that ankle sprains are a global health problem that has a multifactorial background, which often develops into a chronic form and leads to a reduction in the quality of life for all age groups.

Complications among injured patients have determined the need for new management methods. These new methods focus on physiotherapy support, a reduction in pain, an improvement in function during therapy and an acceleration of the return to full physical activity and thus, life activities. In addition, an important aspect within the context of an ankle sprain is the chronicity of injuries to the ankle complex and long-lasting pain. These are not only due to the failure to perform specialist therapy, but also to the lack of optimal treatments and rehabilitation models that are currently being sought, as well as rehabilitation procedures [3].

The kinesiology taping method is often used to alleviate dysfunction and support rehabilitation for chronic diseases [4]. Its usefulness has been demonstrated in the treatment of complications after mastectomy surgery, which were associated with oedema and the excessive accumulation of lymph, as well as in the treatment of other pathologic conditions [5]. Many studies have reported that kinesiology taping aids in the rehabilitation of lower limbs [6,7]. Dynamic taping is an improved version of kinesiology taping that is currently recommended in physiotherapy practice [8], but there is still limited evidence for its effectiveness in the literature. Within this context, the use of dynamic taping for dysfunctions of the ankle complex seems to be important due to the high prevalence of these injuries [1,2].

The essence of kinesiology taping application is to counteract the mobility of the foot, which in turn reduces the patient’s activity until the injury is resolved while, at the same time, setting specific requirements for the material technology of the tape itself. Dynamic taping acts differently, without limiting the movement of the joint and maintaining the ability to perform dorsal and plantar flexion to full extent while fully balancing in the frontal plane [9]. It also allows patients to maintain participation in daily activities at the same time. This is due to different technical parameters of the material, which ensure the elastic properties of the tape. An important effect of using kinesiology tape or dynamic tape is the reduction in limb injuries during competitive sports (especially individual disciplines, such as tennis, dance, athletics, etc.) and team sport games, for which these solutions are proposed not only during the training cycle, but also during competitions [10,11,12,13]. Furthermore, dynamic tape appears to not only limit potential mechanical injuries, but also facilitate locomotion and significantly improve walking speed and stride length for patients with lateral ankle sprains [6].

Ankle sprains are an important public health problem as they lead to temporary absenteeism. Additionally, 20% to 40% of acute ankle sprains progress to chronic ankle instability [14]. Injuries to the ankle joint, together with the knee joint, are among the most common injuries to the musculoskeletal system [15]. Furthermore, ankle sprains are injuries that have a high probability of recurrence, which can cause functional disability; therefore, proper treatment, restorative exercises and improvement in postural stability are extremely important [6].

About 50% of patients who are referred to specialist therapy present with persistent symptoms of chronic joint instability (functional or mechanical), which may lead to recurrent sprains. Mechanical instability manifests itself through excessive joint mobility, which is seen in clinical tests. On the other hand, functional instability is associated with subjective instability symptoms, but not necessarily with concomitant joint laxity. Overall, instability may be associated with recurrent sprains, chronic pain, damage to the cartilage within the joint, the earlier development of degenerative–deforming lesions and the general long-term impairment of performance [15]. Thus, a sustained and rapid improvement in stability is an essential part of recovery. In the literature, the most frequently suggested support for improving stability and balance is the short-term immobilization of the joint using an orthosis, semi-rigid brace or lace-up brace [16,17,18,19], depending on the degree of damage that is seen, as well as a tailored program of physiotherapy that is based on lymphatic drainage techniques, manual therapy or comprehensive exercises to restore nerve and muscle control and balance [20,21,22,23,24].

The method of restoring functionality that is mentioned above has some limitations, which are related to the need to assist in movement, even in the case of everyday activities, and an increased risk of falling; however, with the exception of serious injuries, ankle sprains are generally treated conservatively and not surgically [6].

We hypothesized that the application of dynamic tape would improve postural stability in patients with ankle sprains. The aim of our preliminary study was to assess postural stability on a stable surface among orthopedic trauma patients with inversion ankle sprains after the application of dynamic tape.

## 2. Materials and Methods

### 2.1. Participants

The study was conducted in accordance with the Code of Ethics of the World Medical Association (Declaration of Helsinki) for experiments involving humans. All subjects provided written informed consent to participate in the study. The study was conducted between 2020 and 2021 and included a population of 30 patients (12 females and 18 males; age 25.5 ± 8.0 years; height 179.4 ± 10.9 cm; body weight 78.2 ± 15.6 kg; body mass index 24.13 ± 3.4 kg/m^2^). Patients were being treated for ankle sprains that occurred 7–21 days before the qualification and experiment dates. They were assessed by an experienced orthopedic surgeon through clinical and ultrasound examinations. The clinical examination included an assessment of their ability to walk with a load on the ankle joint, declared pain, swelling and the mobility and stability of the ankle joint. The ultrasound examination assessed damage to the ankle ligaments (no damage, partial tear or complete rupture). Ankle fractures, dislocation and subluxations were excluded in all patients using X-ray images. Patients with grade I and II ankle sprains, according to the American College of Foot and Ankle Surgeons, were enrolled in the study [25]. None of the patients had other diseases of the locomotor system or neurological conditions. Qualification took into account an evaluation of the possibility of undertaking daily physical activity, determining laterality and distinguishing the affected limb.

### 2.2. Evaluation of Parameters and Procedure

Postural stability was assessed on a stabilographic platform (PRO-MED, Legionowo, Poland) [26,27], which was operated with standard Balansis software [28,29].

Balance in a standing position was assessed on the basis of displacements in the projection of the center of pressure (COP) by registering sways during sets of three tests, including tests with open and closed eyes and tests with feedback, which were conducted before and after the application of dynamic tape.

Under static conditions, COP is the projection of the body’s center of gravity onto the support plane [30]. A displacement of the COP point in the frontal plane (*X* axis) corresponds to a right- or left-hand sway while a displacement of the COP point in the sagittal plane (*Y* axis) corresponds to a forward or backward sway. The standard tests on a stable surface consisted of 32-s recordings for each test. The results took into account the data from the XY platform surface, which were related to COP displacements in the XY horizontal plane, at a sampling frequency of 32 Hz and an accuracy of 12 bits when converting the digital signal into an analogue reading. As is already known in the case of the mean radius of sways, the lower the value, the more correct the body posture at the center (XY). On the other hand, with regard to coordination, the higher the value, the greater the ability to maintain the center of gravity along the vertical axis.

The feedback measurement was based on the visual–motor self-control of the COP point, which the patient observed on a screen as a moving black dot against the background of a white square. This enabled the patients to correct their posture immediately. Thus, the patients could evaluate their coordination, i.e., the effectiveness of their sway correction. The value of this parameter, which was given as a percentage, was the average time of the COP being visible and stationary in the white square. This field was a reference point for the subject and allowed for a controlled return to the center (XY) in the case of a deflection. The coordination parameter result was better when the patient presented with fewer deflections and was able to keep the COP point within the white field.

**Figure 1:**
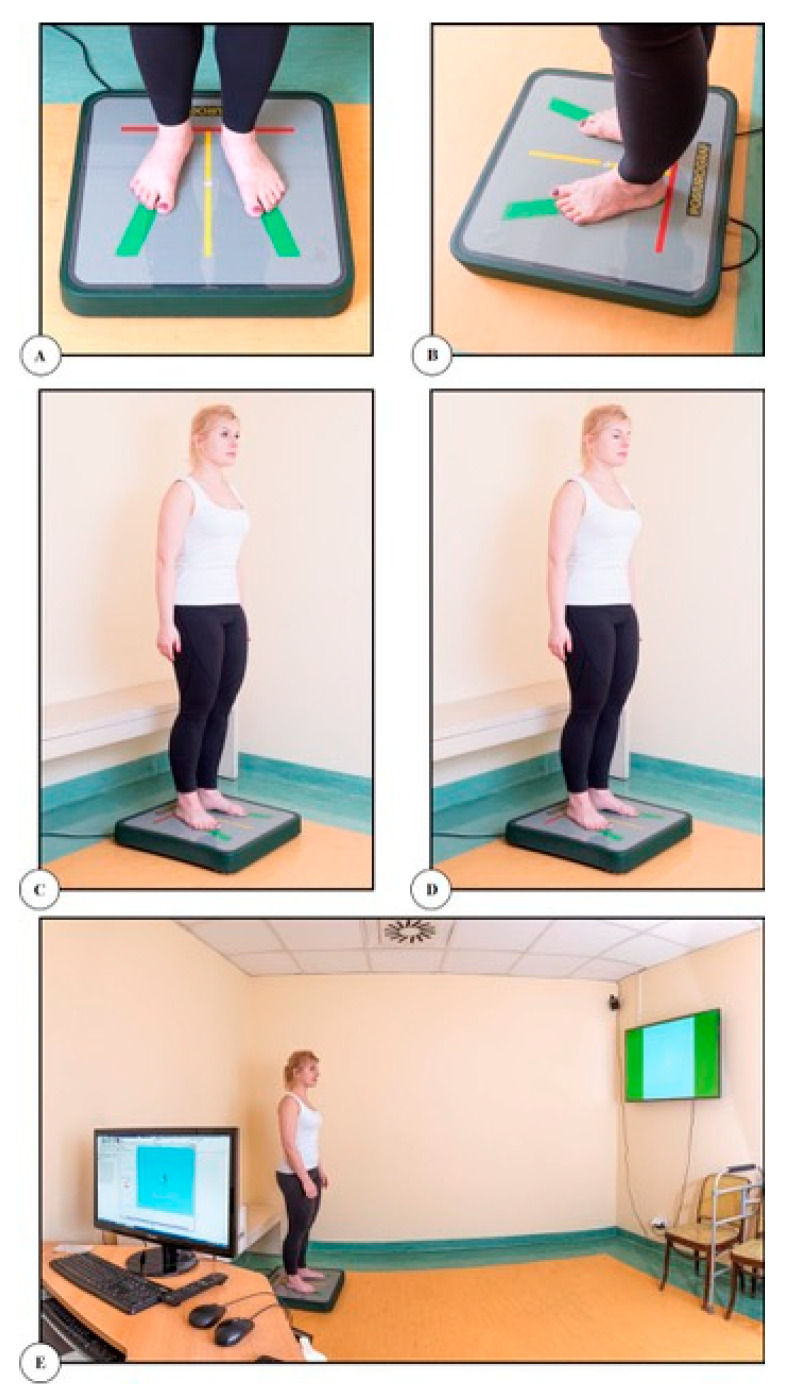
Stability measurement on a stable platform: (**A**) placement of feet (front view); (**B**) placement of feet (side view); (**C**) test with open eyes; (**D**) test with closed eyes; (**E**) test with visual feedback.

The patients were instructed in measurement conduct. The positioning of the feet on the stable surface is shown in Figure 1. The patients were also instructed to keep their arms loosely hanging along their trunk, avoid any movement and breathe freely.

To sum up, the stability measurement consisted of three 32-s tests in a standing position, as shown in Figure 1: one with eyes open, one with eyes closed and one with the so-called feedback, including visual control (visual feedback). The platform was calibrated and centered to the intermediate COP position before and after each test. Each recording started 4 s into the test. The study was carried out using the same procedure before and after the application of dynamic tape, i.e.,

Preliminary examination (test without dynamic tape):

1. Stable surface: eyes open, eyes closed, with feedback;

Final examination (test with dynamic tape):

2. Stable surface: eyes open, eyes closed, with feedback.

In terms of the balance COP, we first analyzed the mean radius of sways (R) and coordination (C) on the stable surface before and after the application of dynamic tape to the injured ankle joint. We then evaluated the differences in the mean radius of sways (R) and coordination (C) before and after the application of dynamic tape to the injured limb.

**Figure 2:**
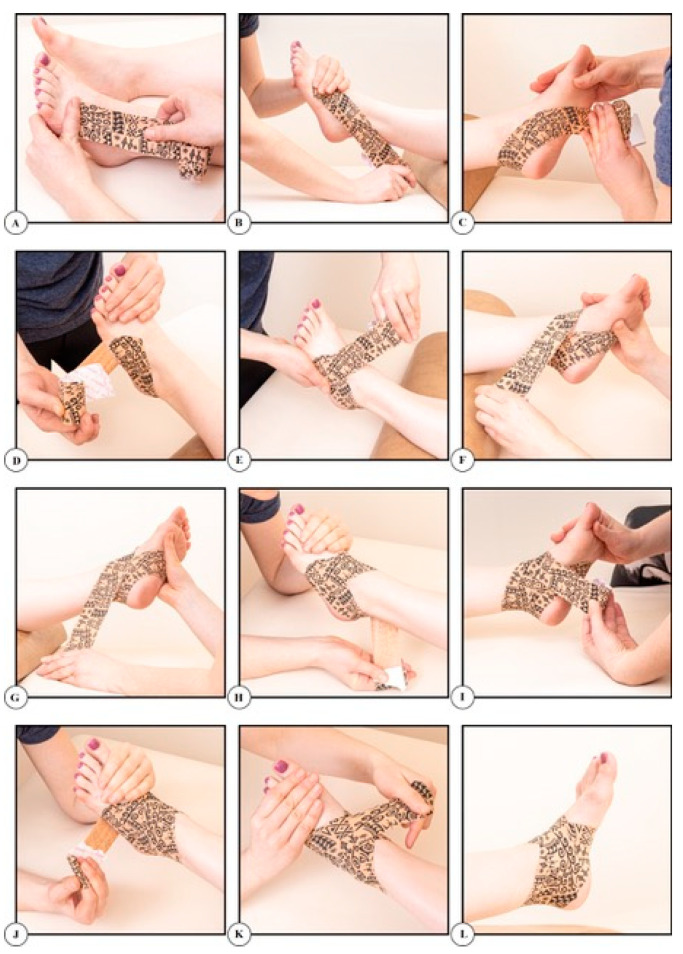
Application of dynamic tape.

After the first two sequences of measurements, dynamic tape (Dynamic Tape, Port Vila, Vanuatu) was applied to the affected joint. During the application of the dynamic tape, the patient was asked to set the foot in the appropriate position (dorsal flexion or plantar flexion) in order to comply with the principles of applying tape to a shortened structure (Figure 2). After the application of the dynamic tape, each patient underwent a 40–45-min adaptation to taping and then, the final examination was carried out on the stabilographic platform.

### 2.3. Statistical Analysis

The statistical analysis was performed using SigmaPlot v.13 (Systat Software Inc., San Jose, CA, USA). The normality of the distribution of variables was tested using the Kolmogorov–Smirnov test with the Lilliefors correction. For the data that did not meet the condition of normal distribution, the differences between the means were analyzed using the Mann–Whitney U test. The significance of the differences between the dependent groups was tested with the Student’s *t*-test for dependent groups (paired *t*-test) or the Wilcoxon signed-rank test (for data that did not have a normal distribution). Differences between the means were considered significant when *p* < 0.05.

## 3. Results

We observed a statistically significant improvement after the application of dynamic tape in both balance and coordination. Regarding balance, the radius of sways (R) decreased from 4.2 ± 1.3 mm to 3.4 ± 0.9 mm (*p* = 0.012) and for coordination (C), the percentage (%) increased from 48.8 ± 19.2 to 59.3 ± 5.8 (*p* = 0.021)

Furthermore, we evaluated the balance (COP) parameters before and after application of dynamic tape for eyes open and eyes closed on a stable surface (Table 1). The following balance (COP) parameters improved after the application of dynamic tape: the mean radius of sways (R); the surface area of the developed trajectory COP (A); the length of the path of the COP point in all planes (L) and its velocity (V) with visual feedback. However, they did not change significantly, both for open and closed eyes (Table 1).

The study also assessed the distribution of load between the healthy limb (unaffected) and the limb carrying the ankle sprain (affected) (Table 2). Load asymmetry was compared both with and without the use of dynamic tape, with eyes open and eyes closed, as well as using visual feedback. All measurements were conducted on a stable surface. Significant differences were only shown for the comparison under the closed eyes condition (34.9 ± 24.4 vs. 41.7 ± 30.5; *p* < 0.01). For the measurements with eyes open and visual feedback, the differences were not statistically significant.

The comparison between the healthy (unaffected) and injured (affected) limbs without dynamic tape showed a significant difference (*p* < 0.05) for open eyes (59.4 ± 29.9 vs. 40.6 ± 29.9 (%)), but not for closed eyes test (53.2 ± 19.9 vs. 46,8 ± 20 (%)) or with the so-called feedback. The same comparison between the healthy and injured limbs with dynamic tape showed positive changes in load distribution for open eyes, but the differences between limbs was also still significant 57.5 ± 28.6 vs. 42.5 ± 28.6 (%) (*p* < 0.05). We did not observe any significant changes after the application of dynamic tape for the tests with closed eyes and feedback (Table 2).

The distribution of load, including the forefoot and heel, was analyzed before and after the application dynamic tape to the injured limb on a stable surface (Table 3). No statistically significant differences were found in the load asymmetry between the forefoot and heel for the tests with eyes open and eyes closed after the application of dynamic tape. The only improvement in the load asymmetry that was close to statical significance was shown for the measurement with visual feedback (*p* = 0.053).

## 4. Discussion

The aim of our work was to evaluate the effectiveness of dynamic taping during selected phases of the rehabilitation process for patients with grade I and II ankle sprains. We limited our preliminary research to the observation of the effectiveness of a 45-min application of dynamic tape to the injured ankle within the context of coordination on a stable surface, which allowed us to search for information on improvements in the effectiveness and support of rehabilitation among this group of patients, including quality of life.

In our study, we assessed the effectiveness of a single application of dynamic tape in improving selected static parameters of postural stability on a stable surface for patients with ankle sprains. In the literature, many studies have focused on the use of kinesiology taping to improve postural stability in both healthy and injured people [4,7,8]. Dynamic taping is a method for improving postural stability. As it was developed as a therapeutic method only a few years ago, it is not yet widely used. We did not find any reports on stability after an application of dynamic tape for patients with ankle sprains. As dynamic tape has a combination of the features of other ankle stabilization techniques, we attempted to refer the results of the use of kinesiology taping to the results that we obtained in this preliminary study.

Other researchers have evaluated the effects of the application of kinesiology tape to the foot and ankle and its possible impact on balance parameters in the case of dysfunctions in the ankle complex [31]. In the study by Jackson et al., balance parameters were assessed in a group of 30 people with chronic ankle instability, which was associated with reduced proprioception and neuromuscular control, using the Balance Error Scoring System. However, balance was evaluated after 48 h of using the kinesiology tape. An improvement in balance was demonstrated compared to a control group with the same dysfunction but without the application of kinesiology tape. It was also confirmed that the balance improvement was permanent, as it was sustained 72 h after the tape was removed. Within this context, it is worth noting that our study showed improvements for a short amount of time after a 45-min experiment with dynamic tape application, although this requires further research. The problem of ankle joint dysfunction and injuries also applies to amateur and competitive sports [32,33]. A study of dynamic balance parameters using the Star Excursion Balance Test (SEBT) in a group of young football players with functional instability of the ankle joint who were treated with kinesiology tape confirmed that the use of kinesiology taping improved dynamic stability in the young football players [32]. The authors compared three groups: participants with therapeutic application, participants with a placebo application and participants without application. In the group of nine people with the therapeutic application, a significant improvement in anterior and posterolateral stability was demonstrated. The change was statistically significant compared to both the placebo group and the group without kinesiology tape [32].

The use of kinesiology tape may also have a positive effect on gait function in addition to balance [33]. In a study on 22 amateur soccer players with lateral ankle sprains, the authors assessed the effects of kinesiology taping on locomotion, including gait parameters (velocity and stride length), and compared people with kinesiology tape, with placebo tape application and without any tape. The players with the proper application of kinesiology tape presented the highest gait parameters. The authors concluded that taping ankle joints is an effective form of therapy and can also be used as an adjunct treatment for this type of injury, similarly to our observations [33]. The cited studies confirmed the positive effects of kinesiology taping [31,32,33], but other reports did not show any improvements in balance parameters after the application of kinesiology taping [34,35]. Hettle et al. examined the effects of various stabilization variants (tests without tape, placebo tape, standard stiff sports tape and kinesiology tape) on balance using functional fitness tests, which were carried out four times at weekly intervals with 16 basketball players who had inversion ankle sprains [34]. In the functional tests, no significant impacts of the use of kinesiology tape or rigid sports tape were demonstrated in terms of an improvement in balance [34]. In the Shields et al. study, 60 physically active school-age participants were divided into a healthy group, coper group and unstable group and examined using the Cumberland Ankle Instability Tool (CAIT) [35]. The tests were conducted before the tape application, immediately after tape application, 24 h after tape application and immediately after the removal of the tape. No significant changes were found after the application of kinesiology tape to the ankle joint [35].

The influence of kinesiology tape on dynamic posture control was also investigated by Nakajima and Baldridge, who assessed its application in 52 healthy people without ankle and lower limb dysfunctions [36]. Subjects were randomly assigned to the experimental group (kinesiology tape with tension) or the control group (kinesiology tape without tension). Dynamic postural control was assessed using SEBT under three conditions: no taping; immediately after taping; and 24 h after taping but with the tape still in place. The results showed a statistically significant effect of the kinesiology tape on dynamic postural control, but they were limited. There was only a significant improvement in the dynamic control of posture in two directions after a 24-h observation in women, with no improvement being shown in men [36].

For application and stabilization, rigid tapes are used in addition to kinesiology taping; however, previous reports have indicated that balance does not improve when using this type of tape [37]. In patients with ankle injuries, a common alternative to kinesiology tape or rigid tapes is the use of soft and semi-rigid orthoses, which can improve balance control and limb loading [38].

We showed that immediately after the application of dynamic tape, the balance and coordination of people with ankle sprains improved significantly on a stable surface. In the study by Kodesh et al. [39], 18 people with a diagnosed chronic instability of the ankle joint were compared to a healthy control group. However, the dynamic tape application was different than that used in our study, as it involved the gastrocnemius muscle. A force plate was also used and the tests were performed with open and closed eyes, similarly to our study. The authors showed that when applied to the calf muscles, dynamic tape significantly improved balance control. There were no differences between people with chronic ankle instability and the healthy people. Dynamic tape improved the balance parameters in all subjects; however, the people with the lowest values of the stability parameters showed a greater improvement in the function of the ankle joint during various physical activities. Thus, dynamic tape was more effective in people with more unfavorable parameters of postural stability. The authors concluded that dynamic tape could be used for people with chronic ankle instability during various life activities to reduce the likelihood of the recurrence of the injury [39].

Improvement in asymmetric load, i.e., the equalization of load between the limbs, is associated with the improved stability of the patient. The lack of significant improvement in our study could have been due to several reasons that affected the symmetry of load. The patient could have experienced pain, which would have led to them relieving the load on the injured foot. Our study could also have been conducted too soon after the ankle sprain. Despite the use of dynamic tape, patients still feared the pain, which was confirmed in an oral interview after the study. In the study by Golec et al. [40], their analysis of the stability of patients with osteoarthritis and osteoporosis showed that each disturbance in the musculoskeletal system reduced stability control and had an impact on the symmetry of load on their feet. In our study on the effectiveness of dynamic tape in improving the symmetry of load, as assessed up to 21 days after the injury, a statistically significant difference was only shown in the case of load asymmetry between the limbs with eyes closed (*p* < 0.01). For the test with eyes open and with visual feedback, the differences were not statistically significant. Furthermore, the results that would have indicated an improvement in the symmetry of load did not improve significantly. This could indicate that visual feedback did not play a significant role in the process of maintaining balance after an ankle sprain or that the use of a stable surface to measure postural stability in laboratory conditions using the eyes open and closed tests with dynamic tape did not have a significant impact on improvements in the tested parameters. However, due to the small size of the group that we evaluated, this research needs to be continued. It is also worth mentioning that when conducting the research, we did not know the conditions of the patients before their injuries.

In other studies that were carried out on patients with neurodegenerative diseases [41,42], significant differences were reported in the mean radius of sways and the surface area and length of the COP path, both for eyes open and closed. In our study, despite observing positive changes, they were not significant. This could indicate that a single application of dynamic tape does not have a significant therapeutic effect. However, this finding requires further study with larger groups. Similar to other physiotherapeutic techniques that are used to assess and improve postural stability, this method requires a demonstration of validity and repeatability, depending on the location of the injury and the type of complication or dysfunction.

Other indications for dynamic tape have also been reported. Robinson et al. [43] used dynamic tape to provide mechanical support and pain relief for patients with greater trochanteric pain syndrome. At the same time, dynamic tape was shown to be effective in improving gait parameters, as it significantly reduced pain and improved mechanical effects. Thus, dynamic tape has a comprehensive effect, which confirms its usefulness for patients with ankle sprains. Our study requires continuation with a larger group, a repeated assessment after a certain amount of time and an investigation into the impact of dynamic tape on pain and quality of life. Another study among football players that assessed pain on the lateral side of the tibia during torsional movements was conducted by Torres et al. [44] proved that the use of dynamic tape reduced pain. Attempts to add dynamic taping to the standard treatment of injuries to the collarbone and acromioclavicular joint also resulted in improved outcomes, so it could be considered as an additional technique to support the physiotherapy process, particularly among children [45,46]. The application of dynamic tape to the shoulder and collarbone complexes contributed to an increase in stabilization and motor control. Ríos [47] evaluated the use of dynamic tape for strains of the biceps femoris muscle. It was concluded that the use of dynamic tape accelerated the process of rehabilitation and the regeneration of the muscle. Lim and Park [8] used dynamic tape for 22 patients with flat feet. They compared the static and dynamic balance of barefoot patients with flat feet both before and after the application of kinesiology taping and dynamic tape. The authors reported that the use of dynamic tape and kinesiology tape increased the patients’ sense of balance. In contrast, dynamic tape showed an advantage in reducing anterior–posterior and lateral variability while walking. The results that have been obtained so far in other injuries and dysfunctions indicate that the use of dynamic tape can increase the effects of physiotherapy.

In therapy and physiotherapy, particularly in the case of ankle joint injuries, the priority is to eliminate swelling and pain and then, to shape and normalize postural stability through the appropriate selection of methods and types of surfaces. Reports from the literature have confirmed that kinesiology taping that is applied to ankle complex can improve not only static but also dynamic balance control [48], while dynamic tape can improve functional performance [49]. On the stable surface in our study, the mean radius of sways changed the most after the application of dynamic tape in the test with eyes open, although the results were not statistically significant. With eyes closed, the mean radius of sways improved as well, but the results were again not statistically significant. In the feedback test, the stability parameters also improved. In the case of the mean radius of sways, a lower value was observed after the application of dynamic tape, which indicated a more correct maintenance of body posture at the center (XY). On the other hand, higher values were found with regard to coordination, which clearly indicated that the studied patients had a greater ability to maintain their center of gravity along the vertical (Y) axis.

To sum up, it can be stated that the use of additional external stabilization in the form of dynamic tape can prove to be helpful during certain tasks, exercises and tests during selected phases of the rehabilitation process, which may lead to an increase in postural stability. At the same time, it has to be remembered that this is merely a supplement to the entire rehabilitation protocol.

Several limitations have to be mentioned for this study. Due to the small number of participants, the study has to be interpreted as a preliminary report and the research will be continued in the future. It is also necessary to include more patients with ankle sprains and to use dynamic tests or tests on unstable surfaces instead of static tests. The application of dynamic tape in everyday life conditions and for longer periods of time than presented here would add considerable value. Another limitation of our work was that it did not take into account gender, age, pain level, level of systematic physical activity or anthropometric parameters, such as body mass index, fat tissue distribution, waist–hip ratio and waist circumference, when assessing balance after The application of dynamic tape.

The unquestioned strength of our study is that, to the best of our knowledge, this is the first study to report the results of using dynamic tape to improve the postural stability of people with ankle sprains. In terms of other rehabilitation methods, dynamic tape seems to be an interesting and perhaps more effective alternative for grade I and II ankle sprains, which requires further research.

## 5. Conclusions

The use of dynamic tape after an ankle sprain significantly improved balance and coordination on a stable surface.

The benefits were shown in terms of a significant improvement in the asymmetric load of the injured limb in comparison to the healthy limb during the test with closed eyes and a considerable improvement in the asymmetric load that was evaluated with visual feedback on a stable surface.

## Figures and Tables

**Table 1:** Results of balance parameters before and after the application of dynamic tape for eyes open and eyes closed on a stable surface.

Parameter	Stable Surface					
	Eyes Open			Eyes Closed		
	Test without DT	Test with DT	*p*-Value	Test without DT	Test with DT	*p*-Value
R (mm)	4.3 ± 1.8	3.9 ± 1.2	0.395	5.1 ± 2.1	4.3 ± 1.3	0.126
A (cm^2^)	515 ± 505	489 ± 271	0.438	757 ± 603	671 ± 491	0.348
L (cm)	373 ± 117	379 ± 96	0.501	476 ± 151	465 ± 133	0.663
V (cm/s)	11.7 ± 3.7	11.9 ± 3.0	0.501	15.0 ± 4.7	14.5 ± 4.1	0.663

A, surface area of the developed trajectory COP; DT, dynamic tape; L, length of the path of the COP point in all planes; R, mean radius of sways; V, mean velocity of the COP trajectory (L/time of recording).

**Table 2:** Load distributions between affected and unaffected limbs on a stable surface.

Load Distribution							
Test	Test without DT			Test with DT			*p*-Value *
	Unaffected Limb (%)	Affected Limb (%)	Asymmetry ^†^	Unaffected Limb (%)	Affected Limb (%)	Asymmetry ^†^	
Eyes Open	59.4 ± 29.9	40.6 ± 29.9	53.7 ± 30.9	57.5 ± 28.6	42.5 ± 28.6	57.6 ± 31.5	0.573
Eyes Closed	53.2 ± 19.9	46.9 ± 20	34.9 ± 24.4	53.1 ± 12.8	46.9 ± 12.8	41.7 ± 30.5	<0.01
Feedback	52.6 ± 14.2	47.4 ± 14.2	25.7 ± 19.0	53.0 ± 25.0	47.0 ± 25.0	24.1 ± 15.5	0.698

DT, dynamic tape; ^†^ load asymmetry between limbs; * value for the difference between the asymmetric load of the limbs before and after the application of dynamic tape.

**Table 3:** Load distributions between the forefoot and heel on a stable surface.

Load Distribution							
Test	Test without DT			Test with DT			*p*-Value *
	Front (%)	Back (%)	Asymmetry ^†^	Front (%)	Back (%)	Asymmetry ^†^	
Eyes Open	51.6 ± 31.4	48.4 ± 31.4	55.0 ± 28.9	41.9 ± 31.0	58.1 ± 31.0	53.9 ± 33.9	0.892
Eyes Closed	43.9 ± 32.0	56.1 ± 32.0	56.5 ± 30.9	37.0 ± 33.2	63.0 ± 33.2	64.3 ± 28.7	0.319
Feedback	54.0 ± 10.9	46.0 ± 10.9	18.3 ± 14.0	49.0 ± 14.5	51.0 ± 14.5	24.2 ± 12.5	0.053

DT, dynamic tape; ^†^ load asymmetry between the limbs; * value for the difference between the asymmetry of load of the limbs before and after the application of dynamic tape.

## Data Availability

The data presented in this study are available upon request.
